# Trigeminal Stimulus Menthol Masks Bitter Off-Flavor of Artificial Sweetener Acesulfame-K

**DOI:** 10.3390/foods11182734

**Published:** 2022-09-06

**Authors:** Kai Büchner, Jana Haagen, Ashtri Sastrosubroto, Roland Kerpes, Jessica Freiherr, Thomas Becker

**Affiliations:** 1 Chair of Brewing and Beverage Technology, Research Group Beverage and Cereal Biotechnology, Technical University of Munich, Weihenstephaner Steig 20, 85354 Freising, Germany; 2 Sensory Analytics and Technologies, Fraunhofer Institute for Process Engineering and Packaging IVV, Giggenhauser Strasse 35, 85354 Freising, Germany; 3 Department of Psychiatry and Psychotherapy, Friedrich-Alexander-Universität Erlangen-Nürnberg, Schwabachanlage 6, 91054 Erlangen, Germany

**Keywords:** *nervus trigeminus*, artificial sweetener, menthol, psychophysiology, off-flavor

## Abstract

Consumer health concerns and regulatory policies lead to a growing demand for sugar-sweetened beverage alternatives. A reduced energy content can be achieved using artificial sweeteners, which often also convey a metallic or bitter off-flavor. Therefore, the alteration of sweetness perception and masking of potential off-flavors are paramount for improving sweet beverages. Trigeminal stimuli, such as capsaicin (spicy) or menthol (cooling), have been used to influence taste perception in food items, although their use in beverages has not yet been systematically investigated. Here, the influence of menthol on sweetness perception in an aqueous solution is examined both on the sensory and psychophysiological level. The addition of menthol had no sensory effect on sweetness perception; however, psychophysiological measurements suggest a boost in the physiological response to cold perception through the addition of sugar. Moreover, menthol addition shifted the recognition threshold of unpleasant bitterness of the sweetener acesulfame-K from 21.35 to 36.93 mg/L, masking the off-flavor. These findings illuminate the complexity of trigeminal perception influences on taste. Further investigation of these effects can render trigeminal stimuli an effective tool to enhance beverage aroma and flavor.

## 1. Introduction

Obesity, showing an increasing prevalence primarily in high-income countries [1], contributes to millions of deaths worldwide [2]. The growing intake of sugar-sweetened beverages, especially among younger individuals [3], is a critical element associated with weight gain and secondary diseases, such as diabetes, stroke, and coronary heart disease [1]. This prompted governments globally to implement regulations and increase awareness of public health [4,5,6]. Sweet sensation is one of the basic tastes; it indicates the presence of carbohydrates, an evolutionary important energy source [7]. In Europe, sucrose is the most commonly used sweetener in beverages [8].

Artificially sweetened beverages emerged as an alternative to sugar-sweetened beverages with lower energy content and similar sensory properties [9]. However, artificial sweeteners often entail bitter or metallic off-flavors [10,11]. These undesirable characteristics [12] negatively affect consumer acceptance of otherwise highly demanded products, such as low-calorie soft drinks or juices [10]. One exemplary artificial sweetener is acesulfame-K, which shows a high sweetness, but simultaneously a pronounced bitter sensation [12]. Consequently, to obtain a well-balanced sensory profile, there is a need to mask these unwanted beverage sensory properties. One possible but barely investigated option to influence the taste of foods or beverages is the use of trigeminal stimulants.

The largest cranial nerve, the trigeminal nerve, is formed by the maxillary, ophthalmic, and mandibular branches. Generally, the trigeminal system conveys information about nociception and chemical irritation and temperature, consistency, and texture of food [13]. Menthol, a trigeminal stimulating substance, naturally occurs in peppermint oil [14]. It activates olfactory receptors in low concentrations, resulting in a minty smell. In medium concentrations, it additionally induces a cooling sensation [15]. By activating transient receptor potential (TRP) channel TRPM8, menthol leads to a higher sensitivity of trigeminal nerve fibers to cold temperatures, and thus enhances cold sensations [14,16]. TRPM8 receptors are present in the trigeminal ganglia and sensory nerve fibers in the tongue [17], enabling the oral-trigeminal effects of menthol. As a molecule with three chiral centers, menthol has eight possible optically active stereoisomers [18], of which the naturally most abundant (1R, 2S, 5R) conformation of L-menthol leads to the strongest cooling sensation [16].

Despite the potential for food aroma optimization, the interaction between menthol and sweetness perception is not yet widely studied. In yoghurt, the addition of menthol showed differing results regarding sweetness perception depending on the age of the tasters [19]. While the addition of menthol overall decreased the perceived sweetness, the younger test group was slightly more sensitive to menthol. On the other hand, splitting the elderly panel into two subgroups according to test performance affected the evaluation of the just-right flavor. One of the more typical uses of menthol as a flavoring agent is the addition to chewing gum. Here, more studies have been conducted, suggesting that fast, high-intensity sweetness release positively influences the menthol perception [20,21]. The influence of menthol and its trigeminal characteristic on the sweetness perception was not investigated. Although the sensory thresholds of mint in liquid matrices have been studied, the trigeminal effect was not considered [22].

Analyzing physiological signals facilitates a deeper understanding of the taster’s reaction toward a product [23]. These psychophysiological measures are applied in product development and marketing since, apart from sensory properties [24], also food-evoked emotions can influence preferences and consumer choices [25]. The sensory evaluations within this study were complemented by psychophysiological measurements recorded via reports and readings. Reports represent users’ subjective experience on a conscious level [25] through self-ratings of physiological and psychological states. Furthermore, measurements of largely unconscious psychophysiological responses were conducted [25,26]. This study includes the conscious rating of different menthol characteristics, sucrose, and mixed samples regarding intensity, familiarity, pleasantness, and arousal (degree of excitement [27]). To assess possible unconscious body responses evoked by the sensory properties of the samples, skin conductance, heart rate (HR), and heart rate variability (HRV) were measured. These parameters are activity measures for the autonomic nervous system (ANS), which can be further divided into the parasympathetic nervous system (PNS) and the sympathetic nervous system (SNS) [28]. While the SNS accelerates the HR under stress, activation or excitation, the PNS slows down the HR in situations of rest or relaxation [26,29]. Therefore, a changing HR can be related to an increase in SNS or PNS activity; an increasing HR is also accompanied by a decrease in vagally-mediated HRV [30]. HRV describes the variation in the interval between successive heartbeats [31]. Higher levels of vagally-mediated HRV at rest are associated with the activation of executive brain functions, and thus with emotional processing and attention [31,32]. The HRV can be analyzed using time-domain, frequency-domain, and non-linear measures [31,32]. While frequency-domain measures are used to determine the relative or absolute amount of signal energy within four frequency bands, time-domain and non-linear measures are used for the quantification of the variability and the predictability of inter-beat intervals, respectively [32].

This study established group thresholds for the perception and recognition of menthol’s trigeminal cooling effect and the sweetness and cooling effects combinations using a modified DIN ISO 3972 protocol. For a deeper understanding of these effects, psychophysiological methods were additionally employed. To evaluate the potential of menthol as a trigeminal stimulant to mask off-flavors, threshold tests were conducted for acesulfame-K bitterness perception in the absence and presence of a steady menthol concentration close to the group recognition threshold.

## 2. Materials and Methods

### 2.1. Sample Preparation

In all samples, Volvic^®^ water (Danone Waters Deutschland GmbH, Frankfurt, Germany) was used. L-menthol was supplied by Destilla GmbH (Nördlingen, Germany), sucrose was obtained from Carl Roth^®^ GmbH + Co. KG (Karlsruhe, Germany), and acesulfame-K was provided by Adelholzener Alpenquellen GmbH (Siegsdorf, Germany). All solutions were tested at 20 °C to avoid influences on the cold perception caused by menthol.

For threshold tests, seven 1000× menthol stock solutions in 40% (*v*/*v*) ethanol were prepared as 2-fold dilution series from a 15.627 g/L menthol stock. Samples and 40% (*v*/*v*) ethanol as control were freshly diluted at 1:1000 for each session. Sucrose thresholds were determined using a concentration gradient from 0.5 to 9.5 g/L in 1.5 g/L steps and pure water as the control sample.

Sucrose influence on menthol recognition was tested with 40% (*v*/*v*) ethanol as control, and menthol stock solutions; all diluted 1:1000 in 3.5 g/L sucrose solution. For menthol influence on sweetness recognition, the sucrose gradient concentrations were dissolved in a 3.45 mg/L menthol solution that was also used as the control sample.

Off-taste of acesulfame-K was investigated with a six-step equivalent sweetness series with 10.0 to 0 g/L sucrose and 0 to 50.0 mg/L acesulfame-K; the same series was also prepared with a constant menthol concentration of 3.45 mg/L.

Psychophysiological measurements were conducted using four samples: 3.907 mg/L pure menthol in water (M in W), 5.0 g/L sucrose in water (S in W), 3.907 mg/L menthol in 3.5 g/L sucrose solution (M in S), and 5.0 g/L sucrose in 3.438 g/L menthol solution (S in M).

### 2.2. Sensory Panels

Panels for most thresholds and all psychophysiological tests consisted of 32 healthy participants, divided into three subgroups of 10, 11, and 11 members. Of those participants, 29 were included in data evaluation. Three participants were excluded due to the fact that they did not correctly identify the stimulus in question. Of the participants included, 21 were female; the mean age was at 27.7 years (SD 8.6 years). Thresholds of artificial sweeteners were tested with 10 participants, 4 female; the mean age was 28.2 years (SD 3.1 years). Inclusion criteria were (i) no allergy to menthol or sucrose, (ii) not being pregnant or breastfeeding at the time, and (iii) being a non-smoker. Tasters were instructed not to eat or drink coffee 30 min prior to the tasting sessions, and the health status was asked beforehand.

In general, panel members were experienced in food and beverage tastings, but unfamiliar with trigeminal stimuli. Therefore, they received a minimum of one training session to get accustomed to menthol in water as a trigeminal stimulus. In between the rated tastings, training sessions were conducted without the initial knowledge of the participants. Here, a random sample concentration was served in duplicate in order that ideally, the participants should not recognize an increase in intensity between the samples. These tests led to the modification of the tasting protocol with defined pauses between sample ingestions. Therefore, the faulty perception of increased intensity due to the additive effect of trigeminal stimulus intensity could be avoided. During the tastings, the participants were asked not only to give a general stimulus threshold, but also to give a recognition threshold with the description or identification of the stimulus in question. Incorrect identification of the stimulus led to exclusion from the test evaluation.

Panel performance was assessed by one-way ANOVA of the three subgroups. The null hypothesis that there is no significant difference in the thresholds between the subgroups could not be discarded with an α-value of 0.05; therefore, there were no significant differences in performance between the panel sub-groups.

All panel members were employees of the institutions conducting the tastings, but not directly involved in the study’s planning and setup. Since only food-grade material was ingested, no ethics committee approval was required.

### 2.3. Test Procedure

Threshold tests were conducted according to a modified DIN ISO 3972 [33] protocol. Modifications of the protocol described below were related to the timing of sample ingestion and sample amount; they were introduced to account for the lingering effect of trigeminal stimuli. Only one trigeminal test per session was conducted with a minimum of 4 h between sessions. All tests were performed in sensory cabins (DIN EN ISO 8589), using digital case report forms. Before each session, tasting instructions were read to the panel where it was specified whether a trigeminal stimulus or a basic taste was sought. The participants wore nose clips and were prohibited from flushing their mouths with water in between the samples. Samples were served as 10 mL volume in 100 mL cups with the total volume to be ingested at once and quickly swirled in the mouth before swallowing. Sensory perceptions were to be noted and rated directly with a consecutive 30 s break before the next sample ingestion. Samples were rated with “0” for no perception, a “?”-symbol for perceivable but not identifiable perceptions, and “X” for recognition. An increasing number of “X”-markings could be given for the identified stimuli with increasing intensity.

**Figure 1:**
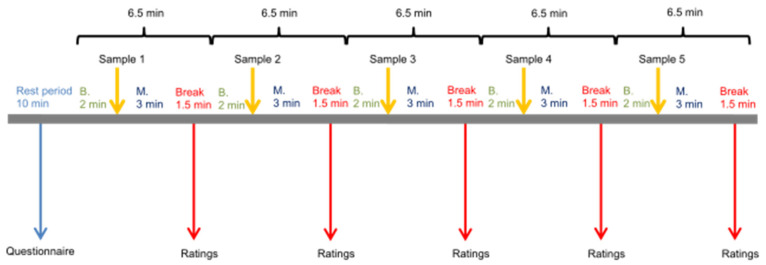
Schematic representation of the psychophysiological test regimen.

Psychophysiological measurements included a 2 min baseline measurement before each sample consumption, a 3 min measuring period after sample consumption, and a 90 s break to rate sample characteristics. Figure 1 shows the tests schedule for the psychophysiological measurements; the same conditions for sample ingestion as in the threshold tests were applied.

The conscious rating was performed on a 100 mm VAS scale reaching from 1 to 101 designed with SoSci Survey (SoSci Survey GmbH, München, Germany). Participants rated the whole sample intensity, menthol intensity, and sucrose intensity from not perceivable to very strongly perceivable. Pleasantness was rated from very unpleasant to very pleasant with the middle of the scale as neutral; familiarity reached from not familiar to very familiar, and arousal was scaled from not arousing to very arousing. Unconscious body responses were acquired via a finger pulse transducer (ADInstruments, Dunedin, New Zealand) and the PowerLab system (ADInstruments, Dunedin, New Zealand). The HRV was analyzed through the standard deviation of the normal-to-normal interval (SDNN) and the square root of the mean squared differences of successive normal-to-normal intervals (RMSSD) as time-domain measures [34] and the absolute power of the high-frequency band (HF power) as a frequency-domain measure [32]. The RMSSD is a measure of short-term variation [34] and can be utilized as an estimate of vagally-mediated changes reflected in HRV [32].

### 2.4. Test Evaluation

The recognition threshold was calculated as the geometric mean of the concentration at which the stimulus was identified and the lower sample concentration before identification. Only the tasters who identified menthol/cooling (n = 18) or sucrose/sweet (n = 23) in both tests were included in the determination of the group recognition thresholds. Group recognition thresholds (RTG) were calculated as the geometric mean of the individual recognition thresholds (RTi). Each group recognition threshold is given with its geometric standard deviation (GSD). Group recognition thresholds were compared as logarithmic sample values [35] using the Wilcoxon signed-rank test, performed in RStudio^®^ (RStudio Inc., Boston, MA, USA) for the comparison of two paired samples at *p* < 0.05 (menthol recognition threshold in water vs. menthol recognition threshold in sucrose solution; sucrose recognition threshold in water vs. sucrose recognition threshold in menthol solution).

Rating data during psychophysiological measurements were analyzed with repeated-measures ANOVA with the different samples set as a factor using Python software [36].

Psychophysiological data were analyzed with LabChart software (ADInstruments, Dunedin, New Zealand). HR in bpm and HRV indicators were determined, i.e., mean normal-to-normal (NN) interval [ms], standard deviation of NN interval (SDNN) [ms], square root of the mean squared differences of successive NN intervals (RMSSD) [ms], and absolute power of the high-frequency band (HF power) [ms2]. All parameters were determined for the 2-min baseline measurement before sample/control consumption (stimulus) and for the 3-min measuring period after consumption; the difference between the before and after stimulus was calculated. A repeated measures ANOVA was performed with the sample and time point (before stimulus vs. after stimulus) set as the factors in Python software [36]. Holm’s test was performed as a post-hoc comparison.

## 3. Results and Discussion

### 3.1. Recognition Threshold

**Figure 2:**
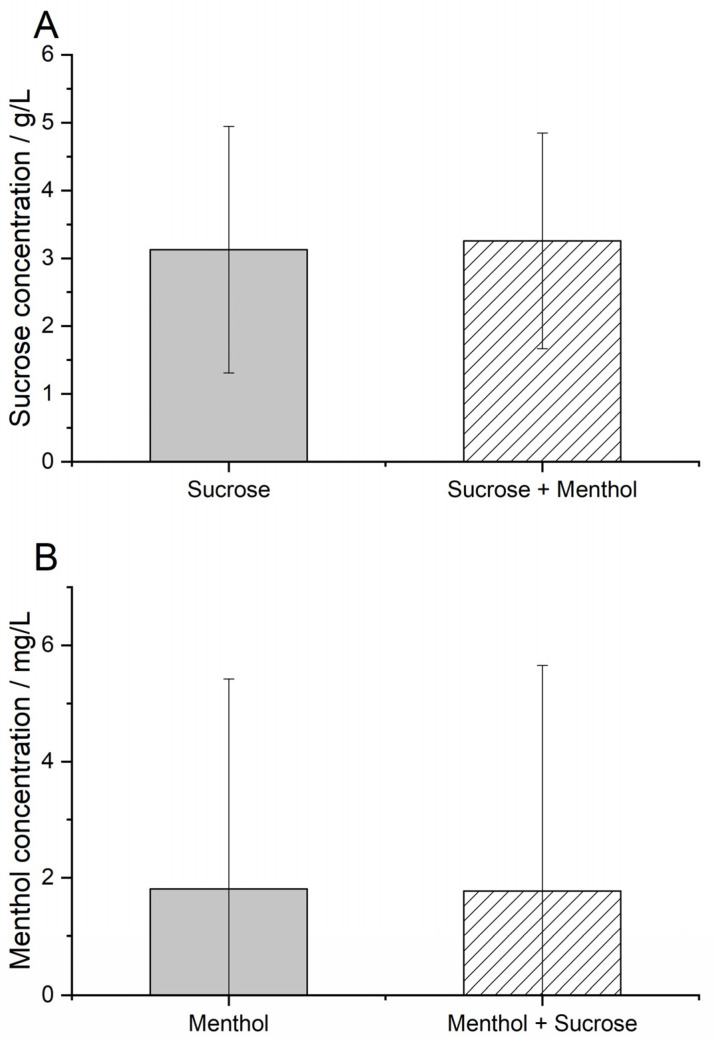
Recognition thresholds for (**A**) sucrose in water and menthol solution, and (**B**) menthol in water and sucrose solution, with geometric standard deviation, rated by n = 32 participants.

Since pre-tests showed a lingering effect of trigeminal stimuli, the DIN ISO 3972 tasting protocol was modified as described in Section 2.3. Taster comments were mandatory for the recognition threshold, enabling the evaluation of the panel’s sensory perceptions. Only participants who clearly identified the stimuli in both sampling pairs were included in the evaluation. For the tasters who clearly identified menthol/cooling or sucrose/sweet in the first sample, this concentration was taken as an individual recognition threshold. In contrast, the actual recognition threshold might be lower. Menthol in concentrations below the recognition threshold was described as pungent (n = 5) or bitter (n = 3). The recognition threshold for sucrose sweetness was not significantly influenced by the addition of a constant concentration of methanol (3.127 g/L, GSD = 1.816 g/L) compared to sucrose in water (3.258 g/L, GSD = 1.589). On the other hand, the addition of sucrose did not significantly affect the recognition threshold for the trigeminal menthol stimulus, as shown in Figure 2; it went from 1.809 mg/L (GSD = 3.611 mg/L) in water to 1.774 (GSD = 3.881) in sucrose solution. The proportion of tasters describing menthol as pungent was even higher (n = 15) than the pure menthol in water samples. In general, tastings for the trigeminal cooling effect showed more widely scattered responses than tastings regarding sweetness. This shows a remaining difference in familiarity for the tasters regarding oral-trigeminal and taste stimuli. However, the geometric standard deviations of the group recognition thresholds also include inter-individual differences between the tasters, such as age, gender, sensory experience or genetic background. Consequently, relative frequency distributions of the individual recognition thresholds are also given.

**Figure 3:**
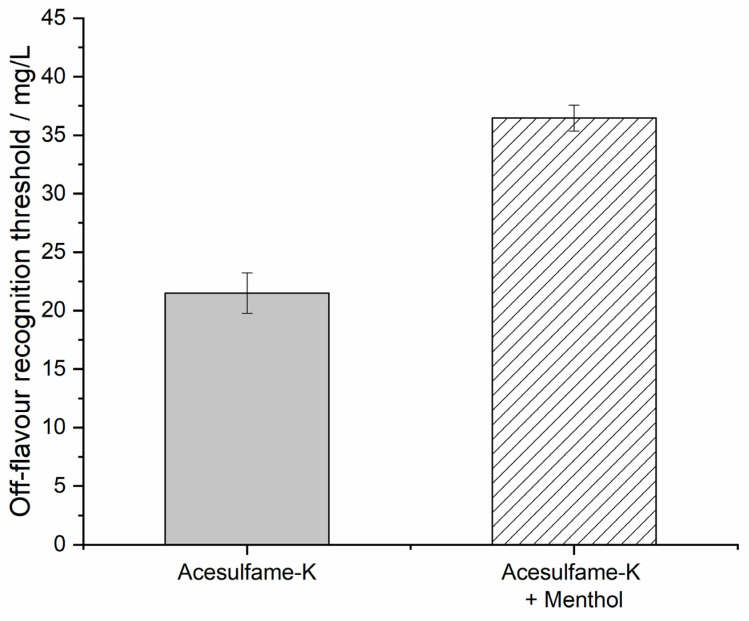
Group thresholds for the off-flavors of acesulfame-K and acesulfame-K in menthol solution with geometric standard deviation, rated by n = 10 participants.

The sensory evaluation of acesulfame-K was focused on the metallic and bitter off-flavors, rated as the intensity of an unknown, non-sweet flavor by 10 tasters. In pre-trials, the threshold test with pure acesulfame-K in ascending concentration did not yield usable results as the growing sweetness of the solution was mistaken by the tasters for the stimulus of interest. More specific formulations of the questionnaire were rejected since they were not leading the tasters to more specific answers, and an influence on the outcome could not be excluded. To overcome this, a test regimen of equivalent sweetness samples was developed. With 10 g/L sucrose as a reference solution, the sweetness of acesulfame-K could be taken out of sensory impressions relevant to the tasting panel. The panel’s comments on the perceived stimulus apart from sweetness ranged from the bitter (n = 4) metallic (n = 3) tastes reported in the literature to astringent, artificial, and plastic-like (each n = 1). The group’s recognition threshold for acesulfame-K’s off-flavor, as shown in Figure 3, was 21.51 mg/L (GSD = 1.73) in the water solution. The addition of a fixed menthol concentration of 3.9 mg/L was able to raise the recognition threshold to 36.45 mg/L (GSD = 1.11). The null hypothesis (H_0_ = the samples with and without menthol addition do not differ in the mean off-flavor recognition threshold) could be rejected, with a = 0.5.

These findings could be practically applied to optimize the taste perception of calorie-reduced beverages. Here, the bitter-metallic taste of artificial sweeteners could be reduced through menthol addition, enabling a potentially higher consumer acceptance. To this end, the menthol concentration should be kept as low as possible to mask the typical smell of the compound. This way, the typical aroma composition of soft drinks, as the consumer expects it, remains unaltered.

### 3.2. Perceptual Ratings

**Figure 4:**
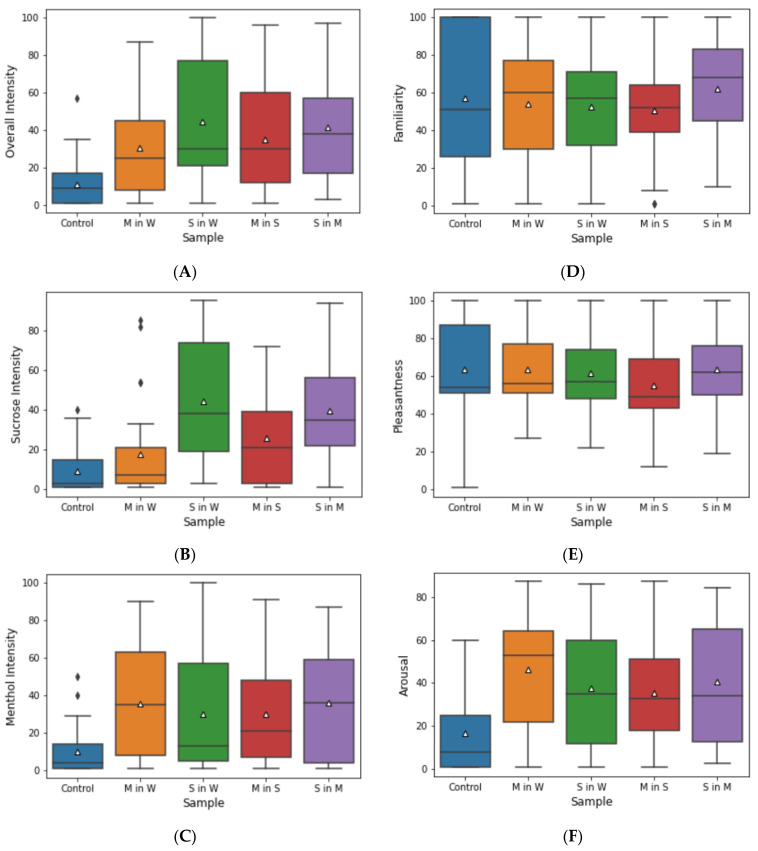
Conscious rating of water (control), menthol in water (M in W), sucrose in water (S in W), menthol in sucrose solution (M in S), and sucrose in menthol solution (S in M). Twenty-nine participants conducted the rating; intensity was rated on a scale from non- to strongly perceptible. All values were consciously rated by the individual tasters. △ symbolize the mean value, ◆ denote outlier values. Overall intensity of all samples (**A**). Intensity of sucrose perception (**B**). Intensity of Menthol perception (**C**). Familiarity of the samples was rated on a scale from not familiar to very familiar (**D**). Pleasantness of the samples, rated from very unpleasant to very pleasant (**E**). Arousal induced by the samples, rated from not arousing to very arousing (**F**).

Conscious rating of the control and the four samples’ overall intensity (Figure 4A) showed a significantly lower intensity rating for the pure water control. The sample M in S was rated as the highest in overall intensity. The overall intensity of this sample was perceived as significantly stronger than pure menthol. Compared to the control sample, sucrose intensity (Figure 4B) was rated as significantly higher in the sucrose-containing sample devoid of menthol than the other samples. While the difference between S in W to the other samples was not significant, this result indicates an effect of menthol on sweetness perception that could not be seen in threshold tests. Menthol intensity ratings exhibit comparable quartile ranges amongst the samples, with the pure sugar in water sample S in W showing a lower, but not significantly lower, median value. The deviation of intensity ratings from the threshold tests might be in part due to the smaller number of samples and the resulting lower accumulation of trigeminal stimuli and the different test settings. All samples were rated as medium familiar, as shown in Figure 4D, with the pure water control scoring the highest, but also showing the widest distributions of quartiles. Remarkably, the pleasantness of the samples, as depicted in Figure 4E, was rated as comparably high with no significant differences. This shows the applicability, especially of the chosen menthol concentrations, since most tasters perceived the trigeminal cold stimulus as at least medium pleasant. Instead, the pure water control was rated as close to very unpleasant in extreme cases. The samples show none of these differences for the maximum values as they were all rated as very pleasant. The arousal rating presents a different distribution of values as shown in Figure 4F. While the pure water control was rated as least arousing, the samples show wide quartile ranges. Holm’s test post-hoc to the one-way repeated ANOVA revealed a significant difference only between the control and the samples. The median value for menthol in water was higher than the other samples, but this difference was most likely caused by the sample being the first sample after the control.

### 3.3. Psychophysiology—Unconscious Body Response

**Figure 5:**
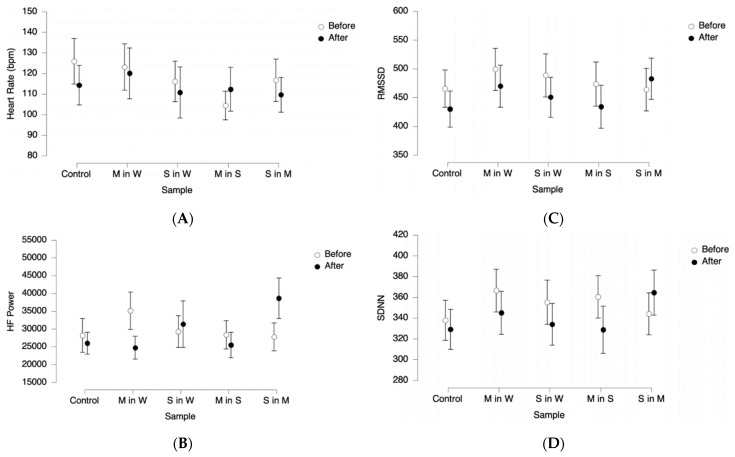
Unconscious responses of 32 participants to samples containing menthol (M in W), sucrose in water (S in W), menthol in sucrose solution (M in S), and sucrose in menthol solution (S in M). Measured responses were heart rate (**A**), the high frequency power band (**B**), the root mean sum of squared distance (**C**), and the standard deviation of the NN interval (**D**).

To assess psychophysiological responses in the tasting panel, a test regimen was developed to minimize the carry-over of trigeminal stimuli between the samples. Figure 5 depicts the unconscious responses of the panel. The pure water control sample was used to investigate whether the drinking process influences the physiological parameters. Notably, the panel’s HR was slightly but not significantly elevated before consuming the control sample. This is most possibly an effect of nervousness due to the unfamiliar situation and the tasters being asked to manage timers and buttons of the experimental set-up themselves. Under mental stress, the hypothalamus releases the corticotropin-releasing hormone (CRH), leading to signals to the brainstem and the spinal cord. This activates the locus coeruleus of the brain stem, thereby increasing the activity of the SNS, reducing the activity of the PNS [37], and accelerating the heart rate. Additionally, mental stress has been linked to low heart rate variability [38,39]. This is supported by the data that suggest that the before-values of all heart rate variability parameters—SDNN, RMSSD, and HF power—were in the lower range for the control compared to the other samples.

Higher levels of vagally-mediated HRV at rest have, among others, been associated with emotional processing and attention [32], while a lower HRV level has been linked to weaker cognitive performance and to emotional arousal [30,31]. In addition, a lower HF power could be further related to anxiety, stress or panic [32].

The high-frequency power band shows differences in the values measured before and after ingestion of the M in W and S in M samples. While this difference was positive for 0.039 mg/L menthol in water (i.e., M in W), consumption of 0.034 mg/L menthol in 5.0 g/L sucrose solution (i.e., S in M) led to higher HF power after ingestion and thus a negative difference. The contrary effect of two samples containing menthol is noteworthy since literature research did not reveal any studies regarding the psychophysiological effects of oral-trigeminal cold-stimuli. A survey regarding the cold pressure test, where a participant’s hands were immersed in iced water, found that the abrupt change in temperature rather than the low temperature itself was the major cause of the sympathetic excitation and parasympathetic withdrawal [40]. A similar effect would suggest that the perceived coolness of the sample in contrast to previous samples would cause the change in HF power. This would explain the reaction to the M in W sample ingested after the control. The two samples that were tested last contained menthol, with the previous sample containing a higher sugar and lower menthol concentration than the previous one. Further investigation is needed into how this would lead to the observed increase in HF power after ingesting the last sample.

To focus on taste and trigeminal perception, all participants wore nose clips to exclude smell perception. This nose clip, allowing only breathing through the mouth, might also affect the physiological parameters since it could be unfamiliar and uncomfortable to the tasters. Since the PNS returns the HR to a normal level in a state of rest or relaxation [26], the first 10 min of the test procedure were used as a designated rest period. In this rest period, the tasters could relax while answering a questionnaire and receiving test instructions.

The heart rate variability parameters SDNN and RMSSD showed a similar behavior, which was expected as both are time-domain measurements for heart rate variability. The graphs indicate a reduction in heart rate variability after the introduction of most stimuli. The decrease in heart rate variability is linked to the increase in heart rate. A study found that the exposure of participants to food increased their heart rate since the cognitive reaction to smell, sight, and taste of the food items affected the activity of SNS and PNS [41]. Nose clips were necessary to exclude any olfactory cross effects on the outcome that were to be expected due to the strong, characteristic smell of menthol.

Furthermore, in contrast to the SDNN data, the decrease in RMSSD was significant, possibly due to the strong influence of the PNS on RMSSD [32]. The increasing heart rate suggests decreasing the PNS activity. Although both SDNN and RMSSD values are based on the activity of ANS, RMSSD is linked more closely to the PNS than SDNN. Moreover, although no significant differences were found in HF power values before and after sample consumption, the samples containing sucrose led to higher after-values than those without sucrose. A study investigating the influence of sucrose ingestion on the hypothalamus and brainstem showed that beverages containing sucrose increased the F power [42]. This is congruent with the sucrose intensity rating (Figure 4), where sucrose-containing samples show a higher distribution and median values than the other samples.

In contrast, menthol intensity was more uniformly rated, even for the sample only containing sucrose in water as shown in Figure 4. This can, despite the training sessions, be explained with the unfamiliarity of the trigeminal cold stimulus in the context of a beverage. In general, the concentration of menthol remained near the threshold determined previously. This was performed considering industrial applications: Higher menthol concentrations might produce more pronounced effects, but also fundamentally alter the characteristics of the beverages used. Therefore, this study regarding the possible effects of trigeminal cold stimuli in beverages were conducted with relatively low menthol concentrations compared to other products, such as chewing gum or toothpaste, leading to indecisive results regarding menthol intensity.

## 4. Conclusions

This study shows interesting effects of menthol as a trigeminal cold stimulus both on conscious taste perception and unconscious body response. Even with the menthol concentration being close to the group threshold in this study, as to not interfere with typical product characteristics in later applications, it shows promising properties as a masking agent since it increased the recognition threshold for acesulfame-K’s bitter off-taste. The acquired data regarding sucrose intensity suggest an interaction of menthol with sweetness perception. Samples containing menthol significantly influenced heart rate variability, suggesting the influence of oral-trigeminal stimulation through menthol on the autonomous nervous system. This study opens an interesting new application for trigeminal stimuli. Further investigation is needed to deepen the understanding of their ANS connection and interaction with the complex sensory matrices of consumer products. In the future, this may enable the use of trigeminal stimuli for masking the artificial sweetener’s off-flavors, and, therefore, a greater acceptance of calorie-reduced drinks by the consumers.

## Data Availability

Data is contained within the article.
